# Mortality and Resource Use Among Individuals With Chronic Kidney Disease or Cancer in Alberta, Canada, 2004-2015

**DOI:** 10.1001/jamanetworkopen.2021.44713

**Published:** 2022-01-25

**Authors:** Marcello Tonelli, Anita Lloyd, Winson Y. Cheung, Brenda R. Hemmelgarn, Matthew T. James, Pietro Ravani, Braden Manns, Scott W. Klarenbach

**Affiliations:** 1 Department of Medicine, University of Calgary, Calgary, Alberta, Canada; 2 Department of Medicine, University of Alberta, Edmonton, Alberta, Canada; 3 Department of Oncology, University of Calgary, Calgary, Alberta, Canada

## Abstract

**Importance:**

Although the public is aware that cancer is associated with excess mortality and adverse outcomes, the clinical consequences of chronic kidney disease (CKD) are not well understood.

**Objective:**

To compare the clinical consequences of incident severe CKD and the first diagnosis with a malignant tumor, focusing on the 10 leading causes of cancer in men and women residing in Canada.

**Design, Setting, and Participants:**

This population-based cohort study enrolled individuals aged 19 years and older with severe CKD or certain types of cancer between 2004 and 2015 in Alberta, Canada. Data were analyzed in November 2021.

**Exposures:**

Individuals were categorized as having severe CKD (based on estimated glomerular filtration rate <30 mL/min/1.73 m^2^ or nephrotic albuminuria without dialysis or kidney transplantation) or nonmetastatic or metastatic cancer (defined by a diagnosis of lung, breast, colorectal, prostate, bladder, thyroid, kidney or renal pelvis, uterus, pancreas, or oral cancer).

**Main Outcomes and Measures:**

All-cause mortality, number of hospitalizations, total number of hospital days, and placement into long-term care were calculated after diagnosis.

**Results:**

Of 200 494 individuals in the cohort (104 559 women [52.2%]; median [IQR] age, 66.8 [55.9-77.7] years), 51 159 (25.5%) had incident severe CKD, 115 504 (57.6%) had nonmetastatic cancer, and 33 831 (16.9%) had metastatic cancer. Kaplan-Meier 1-year survival was 83.3% (95% CI, 83.0%-83.6%) for patients with CKD, 91.2% (95% CI, 91.0%-91.4%) for patients with nonmetastatic cancer, and 52.8% (95% CI, 52.2%-53.3%) for patients with metastatic cancer. Kaplan-Meier 5-year survival was 54.6% (95% CI, 54.2%-55.1%) for patients with CKD, 76.6% (95% CI, 76.3%-76.8%) for patients with nonmetastatic cancer, and 33.9% (95% CI, 33.3%-34.4%) for patients with metastatic cancer. Compared with nonmetastatic cancer, the age-, sex-, and comorbidity-adjusted relative rate of death was similar for CKD (adjusted relative rate, 1.00; 95% CI, 0.97-1.03; *P* = .92) during the first year of follow-up and was higher for CKD at years 1 to 5 (adjusted relative rate 1.23; 95% CI, 1.19-1.26). During the first year of follow-up, for patients with CKD, adjusted rates of placement in long-term care (adjusted relative rate, 0.88; 95% CI, 0.82-0.94) and hospitalization (adjusted relative rate, 0.65; 95% CI, 0.64-0.66) were lower than rates for patients with nonmetastatic cancer; however, those rates were higher for the CKD group than for the nonmetastatic cancer group during years 1 to 5 (long-term care placement, adjusted relative rate, 1.36; 95% CI, 1.29-1.43; hospitalization, adjusted relative rate, 1.55; 95% CI, 1.52-1.58). As expected, adjusted rates of long-term care placement and hospitalization were higher for patients with metastatic cancer than for the other 2 groups.

**Conclusions and Relevance:**

In this study, mortality, hospitalization, and likelihood of placement into long-term care were similar for CKD and nonmetastatic cancer. These data highlight the importance of CKD as a public health problem.

## Key Points

**Question:**

Are the clinical consequences associated with severe chronic kidney disease (CKD) comparable to those associated with cancer?

**Findings:**

In this cohort study of 200 494 individuals, unadjusted mortality among patients with CKD at 1 year and 5 years was higher than mortality among patients with common forms of nonmetastatic cancer.

**Meaning:**

These findings suggest that the risks of mortality and other patient-important outcomes among patients with CKD are comparable to those for patients with nonmetastatic cancer, which highlights the importance of CKD as a public health problem.

## Teaser

This cohort study compares the clinical consequences of incident severe chronic kidney disease (CKD) and the first diagnosis of a malignant tumor among men and women residing in Canada.

## Introduction

Chronic kidney disease (CKD) is a common, potentially treatable condition that is associated with poor outcomes and high health care costs.^1^ Communicating the importance of preventing and managing CKD to decision-makers has been challenging, in part because the clinical outcomes that follow from advanced kidney disease are not well understood.^2^ In contrast, both decision-makers and the general public are aware of the risks of death and disability that are associated with cancer, especially the most common solid malignant tumors.^3^

We designed this cohort study to compare the clinical consequences of incident severe CKD and the first diagnosis with a solid malignant tumor, focusing on the 10 leading causes of cancer in men and women residing in Canada. We used a population-based cohort of all patients with a new diagnosis of severe CKD but who were not receiving kidney replacement therapy (dialysis or a kidney transplant), as well as those with newly diagnosed nonmetastatic or metastatic lung, breast, colorectal, prostate, bladder, thyroid, kidney or renal pelvis, uterus, pancreas, or oral cancer. We hypothesized that mortality, placement in long-term care, and total number of days in hospital would be similar among those with CKD compared with participants with nonmetastatic malignant tumors, albeit lower than those with metastatic malignant tumors.

## Methods

### Study Database

We used a population-based database^4^ that incorporates data from Alberta Health (AH), the provincial health ministry, including demographic characteristics, physician claims, hospitalizations, ambulatory care utilization, the 2 provincial renal programs, and the clinical laboratories across Alberta. More than 99% of Alberta residents are registered with AH and have universal access to hospital care and physician services. This cohort study follows the Strengthening the Reporting of Observational Studies in Epidemiology (STROBE) reporting guideline for observational studies.^5^ The institutional research ethics boards at the Universities of Calgary and Alberta approved the study. The Conjoint Health Research Ethics Board University of Calgary granted a waiver of consent requested by the researcher because the data were deidentified. We used the database to assemble a cohort of individuals aged 19 years and older with severe CKD or certain types of cancer between 2004 and 2015.

### Study Cohort

The cohort was divided into 3 disease groups: severe CKD, nonmetastatic cancer, and metastatic cancer. We excluded individuals who met criteria for CKD or cancer but were receiving renal replacement treatment at baseline. Individuals younger than 19 years were excluded to allow at least 1 year of laboratory, hospitalization, and claims data, which became available at age 18 years (eFigure in the Supplement).

Criteria for incident severe CKD^6^ were as follows: (1) outpatient estimated glomerular filtration (eGFR) less than 30 mL/min/1.73 m^2^ or outpatient albuminuria (nephrotic albumin-to-creatinine ratio >220 mg/mmol or protein-to-creatinine ratio >350 mg/mmol) on or after April 1, 2004, to December 31, 2015, and at least 1 year after the date of registration with AH; and (2) no previous eGFR or albuminuria measurements meeting criteria for CKD. To ensure that all participants had incident rather than prevalent CKD, all were required to have at least 1 previous eGFR measurement greater than 30 mL/min/1.73 m^2^. The date of the first eGFR or albuminuria meeting criteria for entry was used as the index date.

Incident cancers of interest included the 10 leading causes of solid malignant tumors (based on incidence in Canada^7^) in men and women. Criteria for an incident cancer of interest were as follows: (1) a diagnosis of lung, breast, colorectal, prostate, bladder, thyroid, kidney or renal pelvis, uterus, pancreas, or oral cancer in 1 or more hospitalizations or 2 or more claims during 2 years or less using hospital abstracts and physician claims (eTable in the Supplement) on or after April 1, 2004, to December 31, 2015; (2) no history of the 10 cancers of interest; and (3) diagnosis occurring at least 1 year after the date of first AH contact. Ductal carcinoma in situ of the breast, oral carcinoma in situ, and carcinoma in situ of bladder or prostate were excluded.

The type of cancer was assessed using the codes from either the hospitalization or the first of 2 claims that resulted in the cancer diagnosis. Individuals with a nonmetastatic cancer of interest were reclassified as having metastatic cancer if they were found to have a metastatic cancer code within 3 months after their diagnosis with nonmetastatic cancer. Individuals with metastatic cancer of unknown origin were excluded. The date of the first cancer diagnosis that met the eligibility criteria was used as the index date.

For the CKD group, those with a cancer diagnosis (based on the 10 cancers of interest) on or before the index date were excluded. For the cancer group, those with an outpatient eGFR less than 30 mL/min/1.73 m^2^ or nephrotic albuminuria on or before the index date were excluded.

Baseline demographic data (including age and sex) were determined from the AH registry file. We used validated algorithms based on claims and hospitalization data to classify participants regarding the baseline presence of 26 comorbidities.^8^

### Ascertainment of Outcomes

To ascertain outcomes, we monitored participants from their index date until the date of death, outmigration from the province, 5 years after cohort entry, or the study end (December 31, 2016), whichever was shorter. Participants in these cohorts were evaluated for all-cause mortality. In addition, we identified hospitalizations (based on the presence of an admission date from the hospitalizations database^4^ during follow-up). We identified the number of hospitalizations experienced by each individual during follow-up. For each identified hospitalization, we calculated the length of stay on the basis of the days elapsed between the admission and discharge dates. For individuals with multiple hospitalizations, the length of stay was the sum of duration of all hospitalizations. Participants were classified as newly residing in long-term care if during follow-up they were discharged to a long-term care facility after hospitalization, or if we identified 2 physician claims at least 30 days apart for services provided in a long-term care facility; the date of discharge or the date of the first claim, whichever was earlier, was deemed the date on which long-term care began. Cause of death was classified into 5 categories, according to our previous work,^9^ as cardiovascular, infection, cancer, other cause, and unknown cause.

### Statistical Analysis

Baseline descriptive statistics were reported as percentages or medians (IQRs) where appropriate. Our major focus was on comparing patients with severe CKD with those with cancer (as opposed to understanding whether any differences were due to differential age and/or comorbidity), and so our primary analyses were done using unadjusted models.

We used the Kaplan-Meier method to first examine 1- and 5-year survival for each disease group. Next we examined 1- and 5-year survival by disease group and year of cohort entry (2004 to 2006, 2007 to 2009, 2010 to 2012, and 2013 to 2015) to evaluate the possibility of era effects, where outcomes varied within disease groups over time. Within each disease group, we tested for linear trend across the era groups. As a secondary objective, we explored how age, sex, and comorbidity were associated with our main findings by calculating relative rates (and their 95% CIs) using Poisson regression setting nonmetastatic cancer as the referent group. Age, sex, and each of the 26 comorbidities were included as main effects in the model. For our secondary outcomes (placement into long-term care, number of hospitalizations, and length of stay), we calculated unadjusted rates (per 1000 person-days) and adjusted relative rates using the same adjustment variables described already.

The threshold for statistical significance was set at 2-sided *P* < .05. We did statistical analyses using Stata MP statistical software version 15.1 (StataCorp). Data were analyzed in November 2021.

## Results

Of approximately 4.3 million adults residing in Alberta between April 1, 2004, and December 31, 2015, the study cohort comprised 200 494 individuals (104 559 women [52.2%]; median [IQR] age, 66.8 [55.9-77.7] years), including 51 159 (25.5%) with severe CKD (total follow-up, 159 000 years), 115 504 (57.6%) with nonmetastatic cancer (total follow-up, 412 000 years), and 33 831 (16.9%) with metastatic cancer (total follow-up, 68 000 years). Among those with cancer, the majority (77.3%) had nonmetastatic cancer. Median (IQR) ages were 76.5 (64.7-84.5) years for those with CKD, 63.7 (53.7-73.6) years for those with nonmetastatic cancer, and 65.8 (55.6-75.9) years for those with metastatic cancer. Slightly more than one-half of individuals in the CKD and metastatic groups were female; almost one-half of individuals in the nonmetastatic group were female (Table 1). Among individuals with nonmetastatic cancer, prostate cancer (24 088 individuals [21%]) and breast cancer (22 834 individuals [20%]) were the most common, followed by colorectal, lung, and bladder cancer. In contrast, lung cancer accounted for 30% of participants (10 124 individuals) with metastatic cancer. All disease groups had a high burden of comorbidities, with comorbidities least common in the nonmetastatic cancer group and most common in the CKD group (Table 1). A small proportion of individuals (354 individuals [0.7%] in the CKD group, 1316 individuals [1.1%] in the nonmetastatic cancer group, and 234 individuals [0.7%] in the metastatic cancer groups) either left the province or ended registration with the health ministry. Among those with CKD, 4.6% (2353 individuals) went on to develop kidney failure (requiring dialysis or transplant), whereas only 0.1% of those with cancer (179 individuals) developed kidney failure. Among those with CKD, 7.9% (4058 individuals) went on to develop a cancer of interest. Among those with cancer, 4.0% (5970 individuals) went on to develop severe CKD, defined as outpatient eGFR less than 30 mL/min/1.73 m^2^ or nephrotic albuminuria.

**Table 1.** Baseline Characteristics, by Disease Group

Characteristic	Patients, No. (%)		
	CKD (n = 51 159)	Nonmetastatic cancer (n = 115 504)	Metastatic cancer (n = 33 831)
Age, median (IQR), y	76.5 (64.7-84.5)	63.7 (53.7-73.6)	65.8 (55.6-75.9)
Age categories, y			
<40	1945 (3.8)	7895 (6.8)	1544 (4.6)
40-59.9	7242 (14.2)	38 573 (33.4)	10 400 (30.7)
60-79.9	21 671 (42.4)	54 651 (47.3)	16 548 (48.9)
≥80	20 301 (39.7)	14 385 (12.5)	5339 (15.8)
Sex			
Female	28 922 (56.5)	57 289 (49.6)	18 348 (54.2)
Male	22 237 (43.5)	58 215 (50.4)	15 483 (45.8)
eGFR at cohort entry, mL/min/1.7 3m^2^^a^			
<30	44 626 (87.2)	0	0
30 to <45	974 (1.9)	3421 (3.0)	1334 (3.9)
45 to <60	1329 (2.6)	10 265 (8.9)	3247 (9.6)
≥60	4029 (7.9)	75 540 (65.4)	21 119 (62.4)
Missing	201 (0.4)	26 278 (22.8)	8131 (24.0)
Albuminuria at cohort entry^b^			
None or mild	18 286 (35.7)	60 253 (52.2)	14 295 (45.2)
Moderate	6560 (12.8)	6288 (5.4)	2258 (6.7)
Severe	4576 (8.9)	2289 (2.0)	785 (2.3)
Nephrotic	6798 (13.3)	0	0
Missing	14 939 (29.2)	46 674 (40.4)	15 493 (45.8)
Type of cancer			
Bladder	NA	9371 (8.1)	758 (2.2)
Breast	NA	22 834 (19.8)	5522 (16.3)
Colorectal	NA	17 843 (15.5)	7426 (22.0)
Kidney and renal pelvis	NA	4040 (3.5)	911 (2.7)
Lung	NA	15 989 (13.8)	10 124 (29.9)
Oral	NA	7460 (6.5)	2874 (8.5)
Pancreas	NA	3417 (3.0)	2824 (8.4)
Prostate	NA	24 088 (20.9)	1304 (3.9)
Thyroid	NA	5104 (4.4)	1019 (3.0)
Uterine (body, not otherwise specified)	NA	5358 (4.6)	1069 (3.2)
Year of cohort entry			
2004 to 2006	11 082 (21.7)	22 431 (19.4)	7396 (21.9)
2007 to 2009	12 327 (24.1)	27 159 (23.5)	8487 (25.1)
2010 to 2012	13 449 (26.3)	30 967 (26.8)	8915 (26.4)
2013 to 2015	14 301 (28.0)	34 947 (30.3)	9033 (26.7)
Comorbidities			
Median (IQR), No.	3 (2-5)	1 (1-3)	2 (1-3)
Alcohol misuse	2958 (5.8)	4408 (3.8)	1959 (5.8)
Asthma	3071 (6.0)	4171 (3.6)	1173 (3.5)
Atrial fibrillation	11 474 (22.4)	8028 (7.0)	2723 (8.1)
Chronic heart failure	17 858 (34.9)	8388 (7.3)	3121 (9.2)
Chronic pain	10 644 (20.8)	19 367 (16.8)	5368 (15.9)
Chronic pulmonary disease	15 870 (31.0)	22 287 (19.3)	8075 (23.9)
Chronic viral hepatitis B	73 (0.1)	131 (0.1)	44 (0.1)
Cirrhosis	990 (1.9)	449 (0.4)	247 (0.7)
Dementia	6960 (13.6)	4006 (3.5)	1450 (4.3)
Depression	7582 (14.8)	12 855 (11.1)	4052 (12.0)
Diabetes	22 448 (43.9)	19 164 (16.6)	6296 (18.6)
Epilepsy	1206 (2.4)	2199 (1.9)	746 (2.2)
Hypertension	44 491 (87.0)	56 522 (48.9)	17 317 (51.2)
Hypothyroidism	10 166 (19.9)	14 792 (12.8)	4168 (12.3)
Inflammatory bowel disease	941 (1.8)	1654 (1.4)	406 (1.2)
Irritable bowel syndrome	1446 (2.8)	2757 (2.4)	710 (2.1)
Multiple sclerosis	380 (0.7)	947 (0.8)	279 (0.8)
Myocardial infarction	5255 (10.3)	4257 (3.7)	1388 (4.1)
Parkinson disease	1203 (2.4)	1212 (1.1)	351 (1.0)
Peptic ulcer disease	605 (1.2)	499 (0.4)	258 (0.8)
Peripheral vascular disease	3320 (6.5)	2529 (2.2)	945 (2.8)
Psoriasis	701 (1.4)	1013 (0.9)	335 (1.0)
Rheumatoid arthritis	3476 (6.8)	3598 (3.1)	1150 (3.4)
Schizophrenia	846 (1.7)	1194 (1.0)	450 (1.3)
Severe constipation	2183 (4.3)	2689 (2.3)	1349 (4.0)
Stroke or transient ischemic attack	11 624 (22.7)	10 892 (9.4)	3768 (11.1)

Abbreviations: CKD, severe chronic kidney disease; eGFR, estimated glomerular filtration rate; NA, not applicable.

^a^ For individuals with CKD, eGFR refers to either the eGFR that resulted in cohort entry for those who entered because of eGFR or the most recent outpatient eGFR within 1 year before entry (if available) for those who entered because of albuminuria. For individuals with cancer, eGFR refers to the most recent outpatient eGFR within the 1 year on or before cohort entry (if available).

^b^ For individuals with CKD, albuminuria is either the albuminuria that resulted in cohort entry for those who entered because of albuminuria or the most recent outpatient albuminuria measurement within 1 year on or before cohort entry (if available) for those who entered because of eGFR. For individuals with cancer, albuminuria is the most recent outpatient albuminuria measurement within the 1 year on or prior to cohort entry (if available). Albuminuria was categorized as none or mild (dipstick negative or trace, protein-to-creatinine ratio [PCR] <15 mg/mmol, or albumin-to-creatinine ratio [ACR] <3 mg/mmol), moderate (dipstick 1+ for albuminuria, PCR 15-50 mg/mmol, or ACR 3-30 mg/mmol), severe (dipstick ≥2+ for albuminuria, PCR 51-350 mg/mmol, or ACR 31-220 mg/mmol), or nephrotic (PCR >350 mg/mmol, or ACR >220 mg/mmol).

### Survival

**Figure 1.**
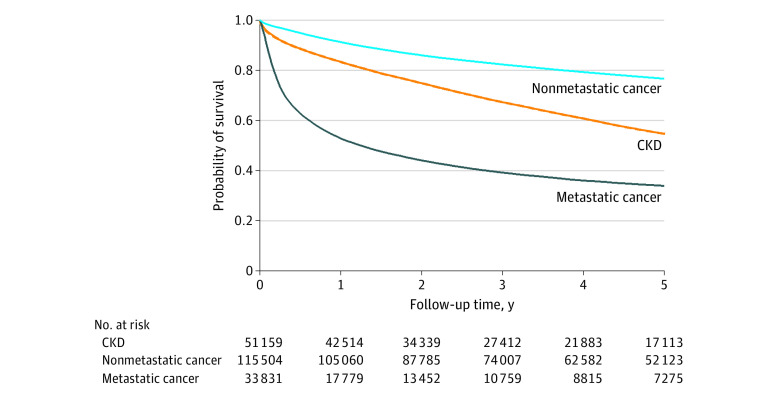
Kaplan-Meier Survival Plot CKD indicates chronic kidney disease.

The Kaplan-Meier survival plot is shown in Figure 1. The Kaplan-Meier 1-year survival was 83.3% (95% CI, 83.0%-83.6%) for patients with CKD and 91.2% (95% CI, 91.0%-91.4%) for those with nonmetastatic cancer and was markedly lower among those with metastatic cancer (52.8%; 95% CI, 52.2%-53.3%). The Kaplan-Meier 5-year survival was 54.6% (95% CI, 54.2%-55.1%) for individuals with CKD, 76.6% (95% CI, 76.3%-76.8%) for individuals with nonmetastatic cancer, and 33.9% (95% CI, 33.3%-34.4%) for individuals with metastatic cancer. The Kaplan-Meier estimates indicated longer 1- and 5-year survival for individuals who entered in later years in comparison to those who entered in earlier years (Table 2), suggesting small improvements over time for those with CKD or nonmetastatic cancer (*P* for trend < .001 for both), but not for metastatic cancer (*P* for trend = .22).

**Table 2.** Kaplan-Meier 1- and 5-Year Survival by Disease Group and Cohort Entry

Survival and cohort entry dates	Patients, % (95% CI)		
	CKD	Nonmetastatic cancer	Metastatic cancer
1-y survival			
2004-2006	81.7 (80.9-82.4)	90.2 (89.8-90.6)	52.2 (51.0-53.3)
2007-2009	82.8 (82.1-83.5)	90.2 (89.9-90.6)	52.6 (51.5-53.6)
2010-2012	83.1 (82.5-83.7)	91.9 (91.5-92.2)	54.2 (53.1-55.2)
2013-2015	85.2 (84.6-85.7)	92.0 (91.7-92.3)	52.1 (51.0-53.1)
5-y survival			
2004-2006	51.9 (51.0-52.8)	74.4 (73.8-75.0)	32.2 (31.1-33.2)
2007-2009	54.4 (53.6-55.3)	75.3 (74.8-75.8)	34.2 (33.2-35.2)
2010-2012	54.9 (54.1-55.8)	77.8 (77.4-78.3)	35.1 (34.1-36.1)
2013-2015	NA	NA	NA

Abbreviations: CKD, severe chronic kidney disease; NA, not applicable.

The age-, sex-, and comorbidity-adjusted relative rates of death were 1.00 (95% CI, 0.97-1.03; *P* = .92) for CKD and 7.52 (95% CI, 7.32-7.73) for metastatic cancer compared with nonmetastatic cancer during the first year of follow-up. Between years 1 and 5 years, the adjusted rate of death was higher for CKD or metastatic cancer (adjusted relative rate, 1.23; 95% CI, 1.19-1.26) than for nonmetastatic cancer (adjusted relative rate, 2.95; 95% CI, 2.85-3.05) (Table 3).

**Table 3.** Unadjusted Rates and Adjusted Relative Rates

Outcome and group	Rate/1000 person-days (95% CI)			
	Unadjusted rate		Adjusted relative rate^a^	
	First year	Years 1-5	First year	Years 1-5
Death				
CKD	NA^b^	NA^b^	1.00 (0.97-1.03)	1.23 (1.19-1.26)
Nonmetastatic cancer	NA^b^	NA^b^	1 [Reference]	1 [Reference]
Metastatic cancer	NA^b^	NA^b^	7.52 (7.32-7.73)	2.95 (2.85-3.05)
New long-term care placement				
CKD	0.14 (0.14-0.15)	0.10 (0.10-0.11)	0.88 (0.82-0.94)	1.36 (1.29-1.43)
Nonmetastatic cancer	0.06 (0.06-0.06)	0.03 (0.03-0.03)	1 [Reference]	1 [Reference]
Metastatic cancer	0.25 (0.23-0.26)	0.05 (0.04-0.05)	4.02 (3.77-4.28)	1.82 (1.67-1.97)
Hospitalizations				
CKD	2.73 (2.69-2.77)	1.68 (1.66-1.71)	0.65 (0.64-0.66)	1.55 (1.52-1.58)
Nonmetastatic cancer	2.98 (2.97-3.00)	0.78 (0.77-0.79)	1 [Reference]	1 [Reference]
Metastatic cancer	7.97 (7.89-8.05)	1.23 (1.20-1.26)	2.65 (2.61-2.68)	1.62 (1.58-1.67)
Length of stay^c^				
CKD	42.27 (41.37-43.18)	26.33 (25.81-26.87)	0.84 (0.82-0.87)	1.65 (1.60-1.70)
Nonmetastatic cancer	28.59 (28.15-29.05)	9.40 (9.22-9.58)	1 [Reference]	1 [Reference]
Metastatic cancer	107.45 (105.66-109.28)	15.03 (14.50-15.58)	3.70 (3.62-3.79)	1.70 (1.63-1.77)

Abbreviations: CKD, severe chronic kidney disease; NA, not applicable.

^a^ Adjusted for age, sex, and comorbidities with the nonmetastatic cancer group set as the referent.

^b^ Unadjusted survival at 1 and 5 years was estimated using the Kaplan-Meier method (see Results section of the text and Table 2).

^c^ For individuals with no hospitalizations, the length of stay was set as 0 days.

### Causes of Death

**Figure 2.**
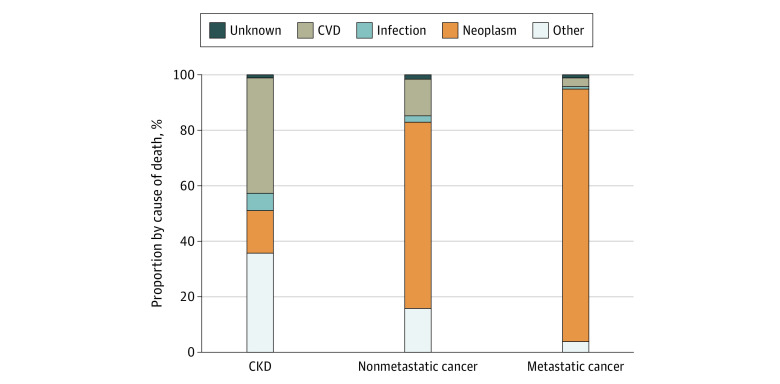
Cause of Death at 5 Years by Disease Group CKD indicates chronic kidney disease; CVD, cardiovascular disease.

The most common cause of death at 5 years among the CKD group was cardiovascular disease. Most patients in the nonmetastatic and metastatic cancer groups died of cancer (Figure 2).

### Long-term Care

Unadjusted rates of placement into new long-term care were highest for patients with metastatic cancer (0.25 per 1000 person-days; 95% CI, 0.23-0.26 per 1000 person-days) compared with patients with CKD (0.14 per 1000 person-days; 95% CI, 0.14-0.15 per 1000 person-days) and those with nonmetastatic cancer (0.06 per 1000 person-days; 95% CI, 0.06-0.06 per 1000 person-days) during the first year of follow-up. Between years 1 and 5, the unadjusted rate was highest for the CKD group (0.10 per 1000 person-days; 95% CI, 0.10-0.11 per 1000 person-days) (Table 3).

During the first year of follow-up, the age-, sex-, and comorbidity-adjusted relative rates of new placement in long-term care were 0.88 (95% CI, 0.82-0.94) for patients with CKD and 4.02 (95% CI, 3.77-4.28) for patients with metastatic cancer compared with those with nonmetastatic cancer. Between years 1 and 5, the adjusted rate of placement into new long-term care was higher for patients with CKD (adjusted relative rate, 1.36; 95% CI, 1.29-1.43) compared with those with nonmetastatic cancer (Table 3).

### Hospitalizations

Unadjusted rates of the number of hospitalizations were highest for metastatic cancer (7.97 per 1000 person-days; 95% CI, 7.89-8.05 per 1000 person-days) compared with CKD (2.73 per 1000 person-days; 95% CI, 2.69-2.77 per 1000 person-days) and nonmetastatic cancer (2.98 per 1000 person-days; 95% CI, 2.97-3.00 per 1000 person-days) during the first year of follow-up. In contrast, unadjusted rates of the number of hospitalizations were highest for CKD (1.68 per 1000 person-days; 95% CI, 1.66-1.71 per 1000 person-days) followed by metastatic cancer (1.23 per 1000 person-days; 95% CI, 1.20-1.26 per 1000 person-days) during years 1 to 5 and were smallest for nonmetastatic cancer (0.78 per 1000 person-days; 95% CI, 0.77-0.79 per 1000 person-days) (Table 3). The age-, sex-, and comorbidity-adjusted relative rates during the first year were 0.65 (95% CI, 0.64-0.66) for CKD and 2.65 (95% CI, 2.61-2.68) for metastatic cancer, compared with nonmetastatic cancer during the first year of follow-up. Between years 1 and 5, the adjusted rate of the number of hospitalizations was higher for CKD compared with nonmetastatic cancer (adjusted relative rate 1.55; 95% CI, 1.52-1.58) (Table 3). Findings for the length of hospital stay were generally similar to those for the number of hospitalizations (Table 3).

## Discussion

In this cohort study using data from a population-based database of more than 4 million adults, we compared clinical outcomes for individuals with severe CKD with those for individuals with nonmetastatic and metastatic cancer. The incidence of severe CKD was substantially lower than that of nonmetastatic cancer. Compared with nonmetastatic cancer, CKD was associated with higher unadjusted mortality over 5 years, predominantly because of deaths due to cardiovascular disease. After adjustment, mortality was similar for CKD compared with nonmetastatic cancer over the first year, but higher over years 1 to 5. CKD was associated with higher unadjusted rates of new placement in long-term care and longer length of hospital stay at 1 year and at years 1 to 5, but after adjustment the higher rates among patients with CKD were observed only for years 1 to 5, whereas patients with nonmetastatic cancer had higher rates of placement and longer length of hospital stay at 1 year. As expected, mortality and hospitalization associated with metastatic cancer were substantially less favorable than that associated with CKD or nonmetastatic cancer, although the latter 2 conditions were much more common.

Tremendous progress has been made in cancer care over the last 2 decades, with rapid developments in diagnosis and treatment that have translated into improvements in clinical outcomes.^10^ This progress is associated with multiple factors, including advances in fundamental science, a strong culture of clinical trials in cancer centers, and the considerable financial benefits that could accrue from successful commercialization of cancer-related medical technologies.^11^ Arguably underpinning all of these developments has been the public perception that cancer is a common and serious condition,^12^ which, in turn, has driven successful fundraising and philanthropic initiatives. In unadjusted analyses, mortality among patients with CKD at 1 year and 5 years was higher than that for patients with nonmetastatic cancer. The total number of hospital days and the likelihood of lost capacity for independent living were both higher among individuals with CKD than for those with nonmetastatic cancer, which accounts for the large majority of cancer cases. The differences between adjusted and unadjusted analyses suggest that the excess risks of long-term care placement or hospitalization were partially associated with age, sex, and comorbidity, especially during the first year. Overall, our findings indicate that the clinical need of patients with CKD is substantial and, arguably, similar to that of patients with cancer. The data presented here may be useful for advocacy efforts that seek to raise awareness about the public health importance of CKD. If such advocacy can be used to strengthen investment in the kidney research agenda, perhaps this would facilitate more rapid progress toward better outcomes for patients with CKD, including better management of CKD-associated comorbidity.

Previous studies^13,14,15,16^ have tended to present the mortality associated with CKD in terms of the excess relative or absolute risk compared with healthy individuals. Although these comparisons are scientifically valid, they may be difficult for the public to understand. Previous studies have compared the mortality associated with kidney failure with that associated with cancer,^17^ typically without stratification for age or cancer type. A recent study^18^ from Ontario, Canada, found that maintenance dialysis treatment was associated with lower survival than several common forms of cancer. Our study extends these findings to the much larger population of patients with severe non–dialysis-dependent CKD and includes data on hospitalization and the likelihood of placement in a long-term care facility. The latter is a proxy for lost capacity for independent living, which is an outcome that is very important to patients and families. Future studies should consider comparing the consequences of non–dialysis-dependent CKD with those associated with other chronic illnesses, such as diabetes, coronary disease, and chronic lung disease.

### Strengths and Limitations

Our study has several important strengths, including its use of population-based data drawn from more than 4 million individuals treated in a geographically defined area served by a universal health care system. Our study also has some limitations that should be considered. First, we used administrative data rather than a prospective cancer registry to identify individuals with cancer. However, to the extent that misclassification in administrative data may be more likely for milder forms of disease, this should have led to overestimates of the apparent mortality associated with cancer and, thus, is unlikely to have affected our conclusions. Second, the results of adjusted vs unadjusted analyses suggest that age and comorbidity likely explain much of the population burden associated with CKD. In addition, the most common cause of death in patients with CKD was cardiovascular disease, whereas most patients in the 2 cancer groups died of cancer rather than comorbidity. Third, to enter the disease group with severe CKD, we required only a single outpatient measurement of either eGFR or albuminuria meeting the criteria threshold, rather than requiring multiple measurements meeting the threshold at a prespecified interval. This approach may have led to misclassification of some individuals, but the potential for such misclassification is greatest at eGFR levels between 45 and 60 mL/min/1.73 m^2^,^19^ rather than levels less than 30 mL/min/1.73 m^2^ as in our study.^20^ In addition, we also required at least 1 previous eGFR measurement greater than 30 mL/min/1.73 m^2^ to increase the likelihood that CKD was incident rather than prevalent. Therefore, we believe that our definition of CKD is unlikely to have affected our conclusions. Fourth, our study is observational and is at risk for residual confounding. Fifth, because CKD is frequently asymptomatic, those who were detected as having CKD may represent a subset of patients with kidney disease who have relatively better access to care, which may have led us to slightly underestimate the risk of adverse outcomes associated with CKD. Sixth, we studied individuals from a single Canadian province, and so generalizability of the findings would require confirmation in other settings.

## Conclusions

In this cohort study, unadjusted mortality at 1 and 5 years was higher among patients with incident severe CKD than among patients with common forms of nonmetastatic cancer. In unadjusted analyses, the total number of hospital days and the likelihood of lost capacity for independent living were both higher among patients with CKD than those with nonmetastatic cancer. After adjustment for age and comorbidity, mortality, rates of placement in a long-term care facility, and rates of hospitalization remained higher for patients with CKD than those with nonmetastatic cancer at 1 to 5 years, although the magnitude of the excess risk was attenuated. These data highlight the importance of CKD as a public health problem.

## Notes

Supplement. **eTable.** Cancer Definitions **eFigure.** Flow Diagram
