# Supplementary material for: Mortality and Resource Use Among Individuals With Chronic Kidney Disease or Cancer in Alberta, Canada, 2004-2015
Source: JAMA Netw Open. 2022 Jan 25;5(1):e2144713. doi: 10.1001/jamanetworkopen.2021.44713 (PMC8790674; doi:10.1001/jamanetworkopen.2021.44713)
Supplement: Supplement. — eTable. Cancer Definitions eFigure. Flow Diagram [file jamanetwopen-e2144713-s001.pdf]

Supplemental Online Content

Tonelli M, Lloyd A, Cheung WY, et al. Mortality and resource use among individuals with chronic kidney disease or cancer in Alberta, Canada, 2004-2015. *JAMA Netw Open*. 2022;5(1):e2144713. doi:10.1001/jamanetworkopen.2021.44713

eTable. Cancer Definitions
eFigure. Flow Diagram

This supplemental material has been provided by the authors to give readers additional information about their work.

© 2022 Tonelli M et al. *JAMA Network Open.*

eTable. Cancer Definitions

**By type**	
Lung<br>ICD-9-CM: 162-163, 231.2<br>ICD-10: C33-C34, C38.4, C45.0, D02.2, C46.71	Non-metastatic
Breast<br>ICD-9-CM: 174<br>ICD-10: C50	Non-metastatic
Colorectal<br>ICD-9-CM: 153-154, 230.3-230.6<br>ICD-10: C18-C21, D01.0-D01.3	Non-metastatic
Prostate<br>ICD-9-CM: 185<br>ICD-10: C61	Non-metastatic
Bladder<br>ICD-9-CM: 188<br>ICD-10: C67	Non-metastatic
Thyroid<br>ICD-9-CM: 193<br>ICD-10: C73	Non-metastatic
Kidney and renal pelvis<br>ICD-9-CM: 189.0, 189.1<br>ICD-10: C64.9, C65.9	Non-metastatic
Uterus (body, NOS)<br>ICD-9-CM: 179, 182, 183.3, 183.4, 183.5, 183.8, 183.9, 233.2, 236.0<br>ICD-10: C54, C55, C57.1, C57.2, C57.3, C57.4, D07.0, D39.0	Non-metastatic
Pancreas<br>ICD-9-CM: 157<br>ICD-10: C25	Non-metastatic
Oral<br>ICD-9-CM: 140-149<br>ICD-10: C00-C14	Non-metastatic
All types<br>ICD-9-CM: 196-199<br>ICD-10: C77-C80	Metastatic

Algorithm: 1 hospitalization or 2 claims in 2 years or less
Ductal carcinoma in situ of breast, oral carcinoma in situ, and carcinoma in situ of bladder or prostate were excluded

© 2022 Tonelli M et al. *JAMA Network Open.*

eFigure. Flow Diagram

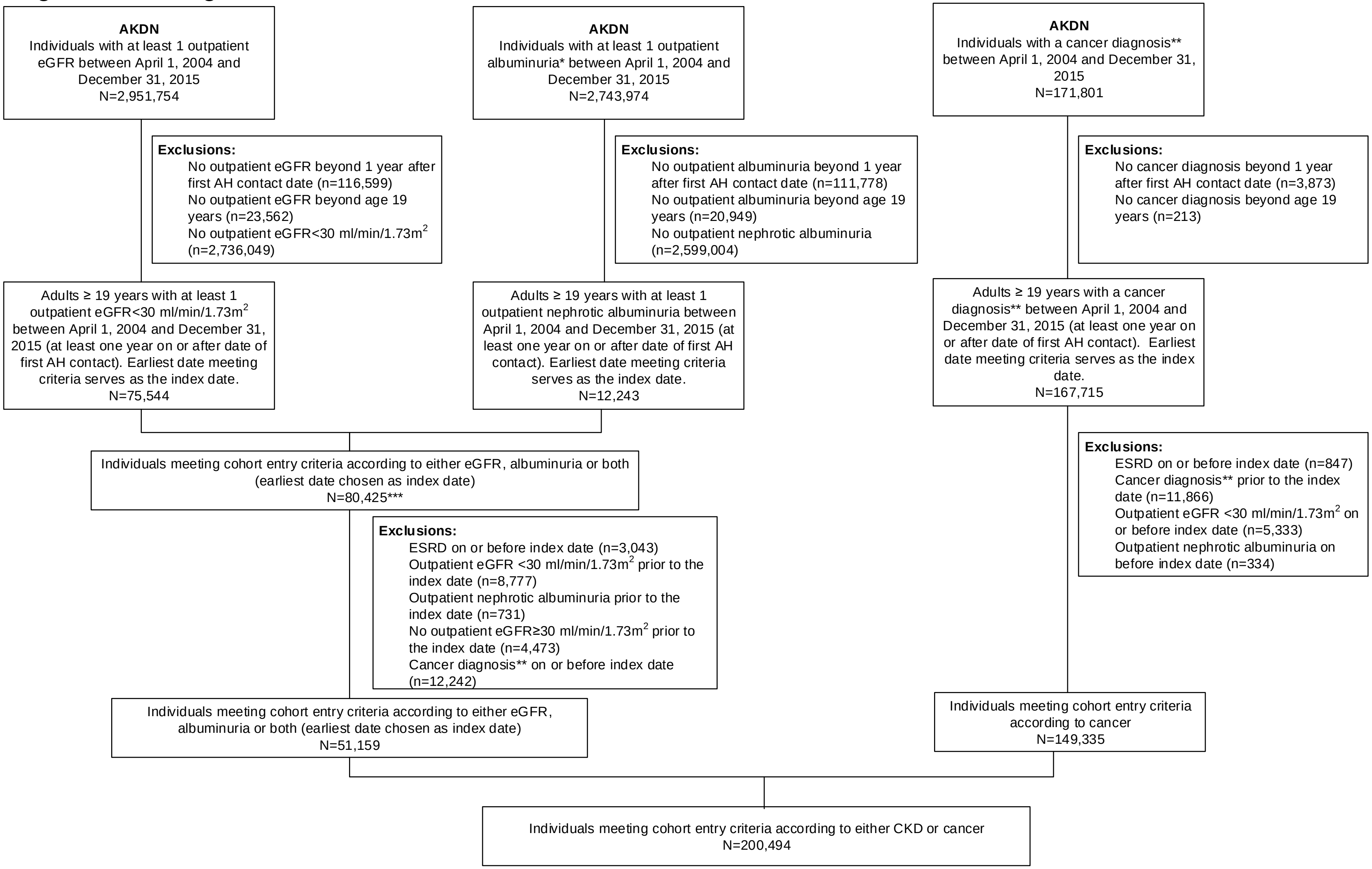

AKDN Alberta Kidney Disease Network, CKD chronic kidney disease, eGFR estimated glomerular filtration rate, ESRD end-stage renal disease
*Albuminuria refers to either albumin:creatinine ratio or protein:creatinine ratio. ** See eTable for cancer diagnosis definition
***At this stage, N=68,182 enter due to meeting criteria for eGFR only; N=4,881 enter due to meeting criteria for albuminuria only; N=567 enter due to meeting criteria for both eGFR and albuminuria on the same date; N=6,795 enter due to meeting criteria for both eGFR and albuminuria on different dates (earlier date chosen with 3,492 having eGFR earlier and 3,303 having albuminuria earlier);

© 2022 Tonelli M et al. *JAMA Network Open.*
